# Evaluation of the Biological Activity of Naturally Occurring 5,8-Dihydroxycoumarin

**DOI:** 10.3390/molecules18044419

**Published:** 2013-04-15

**Authors:** Gražina Slapšytė, Veronika Dedonytė, Juozas R. Lazutka, Jūratė Mierauskienė, Vaidotas Morkūnas, Rita Kazernavičiūtė, Audrius Pukalskas, Petras Rimantas Venskutonis

**Affiliations:** 1 Department of Botany and Genetics, Vilnius University, M.K.Čiurlionis Str. 21/27, LT-03101 Vilnius, Lithuania; E-Mails: grazina.slapsyte@gf.vu.lt (G.S.); veronika.dedonyte@gf.vu.lt (V.D.); juozas.lazutka@gf.vu.lt (J.R.L.); jurate.mierauskiene@gf.vu.lt (J.M.); vaidotas.morkunas@gf.vu.lt (V.M.); 2 Department of Food Technology, Kaunas University of Technology, Radvilėnų pl. 19, LT-50254 Kaunas, Lithuania; E-Mails: rita.kazernaviciute@ktu.lt (R.K.); audrius.pukalskas@ktu.lt (A.P.); rimas.venskutonis@ktu.lt (P.R.V.)

**Keywords:** 5,8-dihydroxycoumarin, antioxidant activity, genotoxicity, chromosome aberrations, micronuclei, sister chromatid exchanges, somatic mutation and recombination

## Abstract

5,8-Dihydroxycoumarin (5,8-DHC) was isolated from aerial parts of sweet grass (*Hierochloë odorata* L.) and screened for antioxidant and genotoxic activities. A clear linear dependency of radical scavenging capacity in DPPH^•^ and ABTS^•+^ assays was determined. 5,8-DHC was very efficient in retarding rapeseed oil oxidation (Oxipress test). TPC (total phenols content) and FRAP (the ability to reduce ferric ion to ferrous ion) assays revealed a somewhat lower antioxidant capacity of 5,8-DHC as compared with gallic acid. Genotoxic activity was tested using different genetic end-points: chromosome aberrations (CAs) and micronuclei (MN) in Wistar rat bone marrow *in vivo*, CAs and sister chromatid exchanges (SCEs) in human lymphocytes *in vitro*, and somatic mutations and recombination in *Drosophila melanogaster* wing cells *in vivo.* 5,8-DHC did not increase frequency of CAs in rat bone marrow cells, but induced a significant increase of MN. It was slightly mutagenic in the *Drosophila melanogaster* assay after 120 h of treatment, but not after 48 h of treatment*.* 5,8-DHC induced both CAs and SCEs *in vitro* in human lymphocytes in a clear dose-dependent manner. Thus, 5,8-DHC may be classified as weakly genotoxic both *in vivo* and *in vitro*.

## 1. Introduction

Coumarins comprise a group of natural phenolic compounds present in a wide variety of higher plants [1]. The coumarins are extremely variable in structure, and till now more than 1,300 structures have been isolated from different natural sources (fruits, vegetables, green tea) and some have been synthesized [2,3]. The total daily human exposure to coumarin (1,2-benzopyrone, the parent compound of coumarin derivatives) from dietary sources together with fragrance use in cosmetic products is reported to be around 0.06 mg/kg/day [1]. Coumarin compounds are also found in a wide spectrum of medicinal plant extracts, and are recognized to possess anti-inflammatory, antiviral, antiallergic, antithrombotic, hepatoprotective, antioxidant, anticarcinogenic activities [4,5,6]. Because of their diverse biological activities and pharmacological properties coumarins have attracted intense interest in recent years.

**Figure 1:**
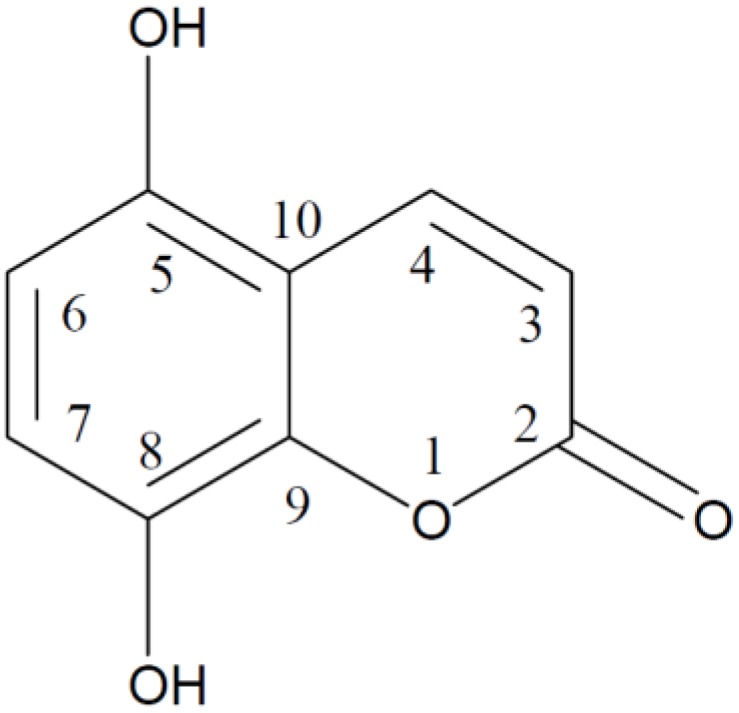
Chemical structure of 5,8-dihydroxycoumarin.

A 5,8-dihydroxycoumarin (5,8-DHC; Figure 1) was isolated from sweet grass (*Hierochloë odorata* L.) in 2002 and it was shown to possess strong radical scavenging capacity [7]. A few years later 5,8-DHC was determined in the chloroform-soluble fraction of the methanolic extract of the whole plant of *Aerva persica* [8]. Due to the presence of 5,8-DHC and its glycoside the extracts from sweet grass were reported as antioxidants, capable of efficiently inhibiting lipid oxidation [9,10,11]. However, cytotoxic properties of 5,8-DHC were reported as well and it was suggested that 5,8-DHC possesses oxidative stress-type cytotoxicity due to the action of its quinodal oxidation product(s) [12].

There is a large body of literature on the toxic, cytotoxic, and carcinogenic potential of coumarin and its derivatives, as well as their protective effects. The most significant hazard of coumarin appears to be liver toxicity, which was demonstrated in mice, rats, dogs, baboons and humans [13] and references therein]. Several studies were conducted to evaluate the genotoxic potential of coumarin, and in general, the available data suggests that coumarin is not a genotoxic agent, at least *in vivo* [14], though a positive effect of coumarin was observed at high doses in bacterial mutation assays or some *in vitro* mammalian tests [15].

It should be noted that the biological activity of coumarins depends upon the various types of substitutions in their basic structure. Unsubstituted coumarins appear to be toxic because of their oxidative decarboxylation, while 4-methylcoumarins are resistant towards oxidative decarboxylation and hence are non-toxic [1]. Coumarins having hydroxyl groups have a more potent protective effect compared to the methoxy-substituted derivatives, and the radical scavenging effects of coumarins are correlated with the number of hydroxyl groups [3,16,17].

Thus, the objective of the present study was to evaluate the biological activity (antioxidant and genotoxic) of the 5,8-DHC, which was not properly tested until now. Antioxidant activity of 5,8-DHC was evaluated by measuring radical scavenging capacity (DPPH^•^ and ABTS^•+^assays), the ability to reduce ferric ion to ferrous ion (FRAP test), total phenols content (TPC test) and rapeseed oil oxidation (Oxipress test). For genotoxicity studies, different genetic end-points were assayed: chromosome aberrations (CAs) and micronuclei (MN) in rat bone marrow *in vivo*, CAs and sister chromatid exchanges (SCEs) in human lymphocytes *in vitro*, and somatic mutations and recombination in *Drosophila melanogaster* wing cells (SMART) *in vivo.*

## 2. Results and Discussion

### 2.1. Antioxidant Properties of 5,8-Dihydroxycoumarin

There are many *in vitro* methods for assessing radical scavenging capacity (RSC) and antioxidant potential of plant origin substances [18]. The RSC measurements in DPPH^•^ and ABTS^•+^ assays, the ability to reduce ferric ion to the ferrous ion (FRAP assay) and total phenols content (TPC) measured by Folin-Ciocalteu reagent are easy, rapid and sensitive methods and therefore the most frequently applied for the preliminary assessment of antioxidant potential of various natural substances.

**Figure 2:**
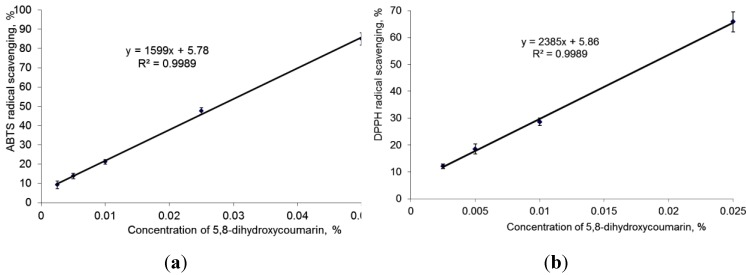
Effect of 5,8-dihydroxycoumarin concentration on radical scavenging capacity. (**a**) ABTS^•+^ assay. (**b**) DPPH^•^ assay.

In our study, a clear linear dependency of radical scavenging capacity for 5,8-DHC in DPPH^•^ and ABTS^•+^ assays was determined (Figure 2). Although the basic principles of these reactions are similar, the ABTS^•+^ scavenging assay is preferable for its ability to evaluate RSC of both lipophilic and hydrophilic antioxidants, and the IC_50_ value of 5,8-DHC in DPPH^•^ assay was 0.0185%, while in the ABTS^•+^ reaction it was remarkably higher, 0.028%.

The Folin-Ciocalteu method is widely used for the TPC assay, although the standard reagent used in this method measures a sample’s reducing capacity [18]. The value measured for 5,8-DHC which is equivalent to the content of total phenols was 581.0 ± 0.0 mg gallic acid equivalents (GAE) in 1 g. This result indicated that 5,8-DHC preparation possessed lower reducing capacity in this reaction as compared with very strong antioxidant gallic acid.

In the FRAP assay, the antioxidant activity is evaluated on the basis of the ability to reduce ferric (III) iron to ferrous (II) iron: a ferric salt (Fe III) is used as an antioxidant and its redox potential (0.70 V) is comparable to that of ABTS^•+^ (0.68 V); therefore, there is not much difference between Trolox-equivalent antioxidant capacity (TEAC) assay and FRAP assay results [18]. The obtained value for 5,8-DHC was 577 ± 0.0 mg/g in Trolox equivalents and it was almost similar to the value obtained with the Folin-Ciocalteu reagent.

**Figure 3:**
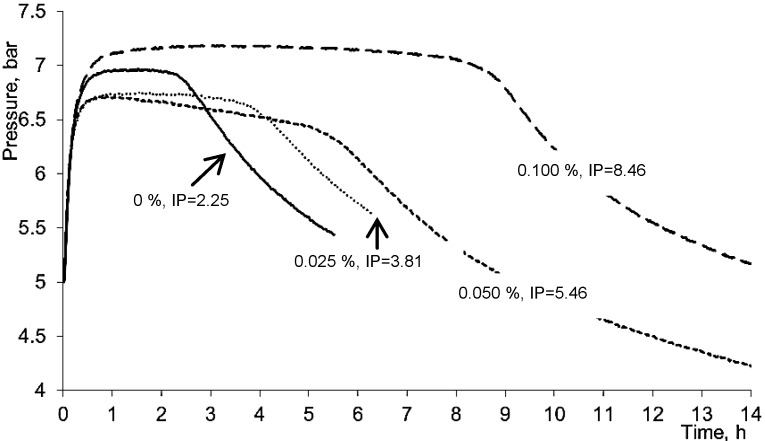
Effect of 5,8-DHC concentration on rapeseed oil autooxidation induction period (IP).

Rapeseed oil contains high percentage of polyunsaturated fatty acids and therefore is very sensitive to auto- and photooxidation. Autooxidation proceeds via radical formation chain reaction, the compounds able to transfer electron and/or hydrogen may retard the process of lipid oxidation. The Oxipress method, which was used to evaluate the antioxidant activity of 5,8-DHC in our study, is a very convenient procedure as it is performed without using any chemicals. The change of oxygen pressure in the reaction vessel at the end of the induction period indicating rapid formation of hydroperoxides can be quite precisely measured. 5,8-DHC was very efficient in rapeseed oil (Figure 3), autooxidation induction period (IP) increased by increasing 5,8-DHC concentration in the oil from 0.025 to 0.1%. Thus, the protection factor of rapeseed, which is achieved by adding 0.025, 0.05 and 0.1% 5,8-DHC, was 1.69, 2.43 and 3.73, respectively.

### 2.2. Genotoxic Potential of 5,8-Dihydroxycoumarin

In the present study, 5,8-DHC was shown to possess strong radical scavenging properties and antioxidant activity. However, it has been demonstrated previously that 5,8-DHC and other polyphenols possess the oxidative stress-type cytotoxic effects due to the polyphenol autoxidation in cell growth media with the production of extracellular H_2_O_2_ [12,19], intracellular generation of reactive oxygen species [20], and depletion of intracellular reduced glutathione [12,21,22]. Some of these processes are known to contribute to genotoxicity of polyphenols [23,24]. Thus, the next objective of the present study was to evaluate genotoxic activity of the 5,8-DHC, which was not tested until now. 

#### 2.2.1. Induction of Chromosome Aberrations and Micronuclei in Rat Bone Marrow Cells *in Vivo*

The results of the CA and MN assays in rats are presented in Table 1. Frequency of aberrant metaphases in blank controls was within the usual range of 1.4–2.0% previously reported in the literature for Wistar male rats [25,26,27]. As expected, animals treated with CP showed a high frequency of abnormal metaphases (21.83 ± 3.25%) , which was comparable with the frequency of 16–21% determined by other authors in rats treated with a single 30–40 mg/kg dose of CP [25,27,28]. The most frequent CAs observed were chromatid breaks and chromatid exchanges (data not shown). In our study, frequency of micronucleated polychromatic erythrocytes (MNPCES) in control animals was lower when compared with the literature data where MNPCE frequencies in the range from 0.33 to 1.5 were reported [27,29]. However, it should be noted that in CP-treated animals we determined an increase of MNPCEs which was proportional to that reported in other studies [27,29,30]. No increase of CAs was determined in animals treated with 5,8-DHC when compared with the vehicle controls. Repeated gavage of rats with 5,8-DHC also had no obvious effect on the frequency of MNPCEs. However, single i.p. injection of animals with 20 mg/kg b.w. of 5,8-DHC resulted in significant increase of MNPCEs when compared with the vehicle controls (0.63 *vs.* 0.38, *p* = 0.001 and 0.59 *vs.* 0.25, *p* = 0.03, in rats sacrificed 24 h and 48 h after dose exposure, respectively). It should be mentioned that vehicle itself as applied intraperitoneally significantly increased frequency of MNPCEs when compared with the blank (untreated) controls. There were no signs of 5,8-DHC cytotoxicity (*i.e.*, no decline of the proportion of PCEs to total erythrocytes). However, sluggishness was observed in all rats receiving 20 mg/kg b.w. of 5,8-DHC at 2–3 h after dose administration and in rats receiving repeated treatment with 10 mg/kg b.w. dose since second dose administration, indicating systemic toxicity of the compound. It should be noted, that in our study i.p injection with 100 mg/kg 5,8-DHC caused sluggishness and death of all treated animals during 3–4 h after administration. This observation prompted us to select the highest dose for the main study as 20 mg/kg b.w., which produced signs of sluggishness, but no deaths.

**Table 1:** Induction of chromosome aberrations and micronucleated polychromatic erythrocytes (MNPCE) in Wistar rat bone marrow cells after treatment with 5,8-dihydroxycoumarin (5,8-DHC).

Treatment group	Sacrifice time (h)	Dose (mg/kg b.w.)	Aberrant metaphases (%± S.E.M.)	MNPCE (%± S.E.M.)	PCE (%± S.E.M.)
Blank control	24	0	1.50 ± 0.27	0.21 ± 0.04	36.4 ± 5.5
Single intraperitoneal treatment					
CP (positive control)	24	30	21.83 ± 3.25 ^a^	1.67 ± 0.22 ^a^	34.8 ± 6.4
Vehicle	24		1.33 ± 0.21	0.38 ± 0.04	30.1 ± 3.4
	48		2.00 ± 0.36	0.25 ± 0.03	36.0 ± 4.4
5,8-DHC	24	10	2.33 ± 0.49	0.56 ± 0.08 ^a^	32.1 ± 1.9
	48	10	1.67 ± 0.56	0.30 ± 0.06	31.8 ± 5.6
	24	20	1.00 ± 0.36	0.63 ± 0.04 ^a^	36.6 ± 2.8
	48	20	2.60 ± 0.68	0.59 ± 0.14^a^	26.6 ± 5.0
Repeated treatment *via* gavage					
Vehicle	48		1.67 ± 0.21	0.24 ± 0.04	48.8 ± 5.1
5,8-DHC	48	3 × 10	1.17 ± 0.17	0.24 ± 0.05	31.7 ± 4.0
	48	3 × 20	2.43 ± 0.37	0.23 ± 0.05	45.5 ± 3.5

^a^ *p* < 0.05 when compared with the vehicle controls.

Numerous previous studies have indicated that coumarin and its derivatives are not genotoxic *in vivo* and do not interact directly with DNA in target organs [31]. Indeed, no induction of MN in mice after coumarin treatment in a dose range of 50–200 mg/kg was observed [14,15,32]. In addition, negative results were reported in an unscheduled DNA synthesis (UDS) test in rats. Edwards *et al.* [33] demonstrated that after oral administration at doses up to the maximum tolerated dose of 320 mg/kg bw, coumarin does not induce UDS in male rat hepatocytes. Moreover, no adduct formation was found in rats after coumarin treatment [13,34], and the antigenotoxic potential of coumarin, probably due to its antioxidant potential, was demonstrated against 7,12-dimethylbenz(a)anthracene-induced DNA damage in the bone marrow cells of golden Syrian hamsters [35]. 

In line to these observations, our data show no increase of CAs in rats treated with 5,8-DHC. However, single i.p. injection of rats with 20 mg/kg b.w. of 5,8-DHC significantly increased the frequency of MNPCEs, which could be probably attributed to the aneugenic effect of 5,8-DHC, since no CAs were induced at the same dose. This proposition is substantiated by the finding of other researches, who demonstrated that coumarin and its derivatives, such as 7-hydroxycoumarin and 4-hydroxycoumarin inhibit mitosis by modifying microtubule dynamics, thus leading to the random distribution of chromosomes at metaphase [36,37]. Future studies distinguishing between micronuclei originating from whole chromosomes (reflect aneugenic effect) and those resulting from acentric fragment (reflect clastogenic effect) should be performed to better elucidate the mode of action of 5,8-DHC.

#### 2.2.2. Induction of Chromosome Aberrations and SCEs in Human Lymphocytes *in Vitro*

In human lymphocytes *in vitro*, 5,8-DHC induced both SCEs and CAs in a dose-dependent manner (Table 2). Since there were no statistically significant differences in the number of 5,8-DHC induced CA frequency between two donors (data not shown), results from two experiments were pooled. Statistically significant increase of CAs was determined after treatment with the highest concentrations of 5,8-DHC (40 μg/mL) only. Dose-response relationship for CAs was clearly linear (R^2^ = 0.81, F = 25.04, *p* = 0.0024). The prevalent type of induced aberrations was chromatid breaks.

**Table 2:** Effects of 5,8-dihydroxycoumarin (5,8-DHC) on the frequency of chromosome aberrations (CAs), sister chromatid exchanges (SCEs) and replication index (RI) values in human lymphocyte cultures *in vitro.*

Treatment	Concentration (µg/mL)	CA per 100 cells ^a^ (±S.E.M.)	Donor A		Donor B	
			SCE/ cell ± S.E.M.	RI ± S.E.M	SCE/ cell ± S.E.M.	RI ± S.E.M
Blank		1.5 ± 0.9	9.9 ± 0.5	2.72 ± 0.04	8.7 ± 0.4	2.45 ± 0.05
Ethanol	7.5 µL/mL	3.0 ± 1.2	10.6 ± 0.4	2.76 ± 0.04	9.5 ± 0.5	2.46 ± 0.05
MMS	0.02 µL/mL	12.0 ± 3.2 ^b^	31.7 ± 1.2 ^b^	2.21 ± 0.05 ^b^	46.7 ± 1.9 ^b^	2.10 ± 0.06 ^b^
5,8-DHC	10	1.5 ± 0.9	10.2 ± 0.5	2.70 ± 0.04	10.1 ± 0.5	2.22 ± 0.05 ^b^
	15	3.0 ± 1.2	12.3 ± 0.6 ^b^	2.74 ± 0.31	10.6 ± 0.5	2.35 ± 0.05
	20	3.5 ± 1.3	12.7 ± 0.7 ^b^	2.65 ± 0.04 ^b^	10.3 ± 0.4	2.49 ± 0.05
	25	6.5 ± 1.7	13.6 ± 0.6 ^b^	2.66 ± 0.04 ^b^	9.9 ± 0.5	2.31 ± 0.05 ^b^
	30	7.0 ± 1.8	13.9 ± 0.6 ^b^	2.55 ± 0.05 ^b^	11.8 ± 0.5 ^b^	2.31 ± 0.06 ^b^
	35	7.0 ± 1.8	15.0 ± 0.8 ^b^	2.51 ± 0.05 ^b^	11.5 ± 0.6 ^b^	2.17 ± 0.06 ^b^
	40	9.5 ± 2.1 ^b^	19.7 ± 1.5 ^b^	2.62 ± 0.04 ^b^	13.3 ± 0.6 ^b^	2.07 ± 0.06 ^b^

^a^ Pooled data of two experiments; 200 metaphases scored per each experimental point; ^b^ *p* < 0.05 as compared to solvent (ethanol) control.

5,8-DHC induced SCEs in lymphocytes of both donors, however, their sensitivity to this compound was different. In the case of donor A, statistically significant increase in the frequency of SCEs was observed at the dose of 5,8-DHC as low as 15 μg/mL, while in the case of donor B statistically significant increase was observed at doses of 30 μg/mL and above. In both cases dose-response relationship was linear (R^2^ > 0.7), however, regression coefficient for the donor A was 2.6 times higher than for the donor B (1.15 and 0.44, respectively), indicating higher sensitivity of donor A to 5,8-DHC as compared to donor B (*p* = 0.008, Wilcoxon signed rank test). Treatment with 5,8-DHC at concentration higher than 25 μg/mL consistently inhibits cell replicative kinetics in lymphocytes of both donors, that may indicate [38] cytotoxicity at these concentrations.

In summary, only a slight (though significant at the highest concentration) increase of CAs was determined in human lymphocytes treated with 5,8-DHC *in vitro*. 5,8-DHC revealed to be more potent inducer of SCEs. A number of *in vitro* genotoxicity studies have been conducted with coumarin, and both negative and positive responses have been reported. A concentration-dependent increase in coumarin–induced CAs was determined in Chinese hamster ovary (CHO) cells in the presence, but not in the absence of metabolic activation by S9-mix from Aroclor 1254-treated rats at the dose range of 50–1,600 μg/mL. In contrast, induction of SCEs was observed in the absence of metabolic activation, but not with metabolic activation [39]. Sasaki *et al.* [40,41] found no evidence of increase in SCE or chromosome aberration in cultured CHO cells treated with coumarin and umbelliferone (7-hydroxycoumarin) at doses up to 333 μM. In our experiments, we found significant inter-donor variability in the number of 5,8-DHC-induced SCEs, indirectly confirming the importance of metabolism for the genotoxicity of 5,8-DHC. 

#### 2.2.3. Somatic Mutation and Recombination Test (SMART) in *Drosophila Melanogaster in Vivo*

The pooled data from two independent experiments are reported in Table 3. Water controls showed the background frequencies of 15.2–13.7% wings with spots and 0.18–0.15 total spots per wing. These values are within the usuall range from 0.15 to 0.50 previously reported in the literature [42,43,44]. The predominant type of spots in water controls was single spots (0.13 spots per wing), while large single spots and twin spots were rare with the frequency not exceeding 0.03 spots per wing. In the positive controls (treated with 50 μM MMS), 100% wings with spots and 7.15 total spots per wing were induced. MMS induced increase in all categories of spots: small single spots (1.65), large single spots (4.63) and twin spots (0.88). The frequencies of wings with spots, total spots and different categories of spots in ethanol controls did not significantly differ from those in the water controls. No statistically significant difference between concurrent controls and the different concentrations of the test solutions was found after 48 h treatment period. However, after 120 h of treatment, statistically significant increase in the number of wings with spots (at concentrations of 0.25% and 0.5%) and mean number of total spots per wing (at all concentrations of 5,8-DHC tested) were observed. It should be noted that 5,8-DHC induced mainly small single spots (range from 0.20 to 0.23 spots per wing). However, increase, though insignificant, of large single spots (0.10 spots per wing) was observed after treatment with 0.5% 5,8-DHC. No increase of twin spots was found (frequency of twin spots was in the range of 0–0.04 spots per wing).

**Table 3:** Summary of results in the *Drosophila* wing spot test after the treatment with 5,8-dihydroxycoumarin.

Concentration of test solution (%)		Exposure duration 48 h			Exposure duration 120 h		
		No. of wings	Wings with spots (% ± S.E.M.)	Spots/wing (±S.E.M.)	No. of wings	Wings with spots (% ± S.E.M.)	Spots/wing (±S.E.M.)
Water		132	15.2 ± 3.1	0.18 ± 0.03	95	13.7 ± 3.5	0.15 ± 0.03
Ethanol (5%)		139	17.3 ± 3.2	0.19 ± 0.03	118	13.6 ± 3.1	0.14 ± 0.03
5,8-DHC	0.1	97	26.8 ± 4.5	0.27 ± 0.04	98	22.4 ± 4.2	0.25 ± 0.04 ^a^
	0.25	90	21.1 ± 4.3	0.22 ± 0.04	96	27.1 ± 4.5^a^	0.30 ± 0.04 ^a^
	0.5	96	25.0 ± 4.4	0.27 ± 0.04	97	28.9 ± 4.6^a^	0.32 ± 0.04 ^a^

^a^ *p* < 0.05 as compared to solvent (ethanol) control

Thus, SMART in *D. melanogaster* demonstrated slight genotoxicity of 5,8-DHC after 120 h treatment, and no statistically significant increase in the number of somatic mutations after 48 h treatment. This difference in effect may be explained by more prolonged time of exposure, which can result in higher frequency of spots. The exposure duration of 120 h covered the evolutionary period from fly egg till case-worm. In this case larvae are not only exposed for a more prolonged time, which can result in higher frequency of spots, but treatment starts with younger larvae when compared with the 48 h treatment protocol (exposure starts when larvae are already 72-h-old). A correlation between the time of induction of genetic damage in the somatic cells and the size of the resulting spot is determined, and large spots are observed after treatment of very young larvae [45,46]. In our study we observed increase of large single spots after 120 h treatment. It could be concluded that 5,8-DHC under certain experimental conditions, *i.e.*, more prolonged treatment time, may reveal slight genotoxic avtivity.

## 3. Experimental

### 3.1. Isolation of 5,8-Dihydroxycoumarin

5,8-DHC was isolated from aerial parts of sweet grass (*Hierochloë odorata* L.). A detailed procedure for 5,8-DHC isolation and its identification were described previously [7,47]. Briefly, dried and ground plant material was extracted with methanol/water/acetic acid (80:20:1) and concentrated in a rotary evaporator. The remaining solution was diluted with ultrapure water and then successively extracted with hexane, *tert*-butyl methyl ether, and finally butanol. The remaining aqueous phase was freeze-dried. The *tert*-butyl methyl ether fraction was loaded on a 50 g silica gel column and eluted with a mixture of hexane/ethyl acetate (1:1). Fractions containing high purity 5,8-DHC were combined, solvents were evaporated in a rotary evaporator and a yellow crystalline material with m.p. of 216 °C has been obtained. The purity of 5,8-DHC based on HPLC-ESI-MS analysis peak area was 93.5%. 

### 3.2. Assessment of Antioxidant Potential

#### 3.2.1. DPPH^•^ Radical Scavenging Assay

Radical scavenging activity of extracts against stable DPPH^•^ (2,2-diphenyl-2-picrylhydrazyl hydrate, Sigma-Aldrich Chemie, Steinheim, Germany) was determined spectrophotometrically by using slightly modified method of Brand-Williams *et al.* (1995) [48]. 5,8-DHC was dissolved in methanol at 0.0025, 0.005, 0.010 and 0.025% concentrations before reaction. A 2 mL aliquot of DPPH^•^ solution in methanol (6 × 10^−5^ M) was mixed with a 50 μL of 5,8-DHC solution in 1 cm path length quartz cuvette. The decreasing absorbance at 515 nm was recorded on a UV spectrophotometer Spectronic Genesys 8 (Spectronic Instruments, Rochester, NY, USA) during 16 min reaction time at 1 min intervals until the absorbance curve reached the plateau. The same amount of methanol and DPPH^•^ solution was used as a blank. The experiments were carried out in triplicate. The capacity to scavenge the DPPH^•^ is expressed as % inhibition, which was calculated using the following formula:

100 × (A_X_ − A_B_)/A_B_ (1)

where A_B_ is the absorbance of the blank sample (t = 0) and A_X_ is the absorbance of the reaction solution at t = 16 min. The results were also expressed as an effective concentration IC_50_, which shows the amount of 5,8-DHC required to decrease the initial DPPH^•^ concentration in the reaction mixture by 50%.

#### 3.2.2. ABTS^•+^ Radical Cation Decolorization Assay

The experiments were carried out using a slightly modified 2,2’-azinobis (3-ethylbenzothiazoline-6-sulfonic acid) diammonium salt (ABTS, Sigma-Aldrich Chemie, Steinheim, Germany) decolorization assay [49]. Stock solution of ABTS (2 mM) was prepared by dissolving in 50 mL of phosphate buffered saline (PBS) obtained by dissolving 0.27 g of KH_2_PO_4_ (Jansen Chimica, Beerse, Belgium), 1.42 g of Na_2_HPO_4_, 8.18 g of NaCl, and 0.15 g of KCl (Merck, Darmstadt, Germany) in 1 L of distilled water. The pH was adjusted to 7.4 using NaOH solution. The ABTS^•+^ was produced by reacting 50 mL of ABTS stock solution with 200 μL of K_2_S_2_O_8_ solution. The ABTS^•+^ solution was diluted with PBS before measurements to obtain the absorbance of 0.800 ± 0.030 at 734 nm. 5,8-DHC was dissolved in PBS at the concentrations of 0.0025, 0.005, 0.01, 0.025 and 0.05% before the reaction. A 3 mL aliquot of ABTS^•+^ solution was mixed with 30 μL of 5,8-DHC solution in the 1 cm path length quartz cuvette. The absorbance was read at ambient temperature after 16 min. PBS solution was used as a blank. All determinations were carried out in triplicate. Radical scavenging capacity was expressed as in DPPH^•^ scavenging assay. 

#### 3.2.3. Evaluation of Total Phenolic Compounds (TPC)

The content of TPC was determined by the Folin–Ciocalteu method [50]. Calibration curve was prepared by using 1 mL reference gallic acid solutions in ethanol (0.025, 0.075, 0.100, 0.175 and 0.350 mg/mL), which were mixed with 5 mL of a standard Folin-Ciocalteu reagent and diluted with distilled water (1:10) and 4 mL of 7.5% sodium carbonate solution in distilled water. The absorption was read after 30 min at 765 nm. The concentration of TPC was expressed in mg of gallic acid equivalents (GAE) per 1 g. The TPC was calculated by the following formula:

C = c × V/m
(2)

where C is the concentration of gallic acid, determined form the calibration curve (mg/mL); V is the volume of 5,8-DHC solution (100 mL); m is the weight of 5,8-DHC (g).

#### 3.2.4. Ferric Reducing/Antioxidant Power (FRAP) Assay

The ability of antioxidants to reduce ferric ion to the ferrous ion, (FRAP assay) is another indicator frequently used for assessing antioxidant power [51]. Ferrous ion (Fe^2+^) produced in this assay forms a blue complex (Fe^2+^/TPTZ) absorbing at 593 nm. Briefly, the reagent was prepared by mixing acetate buffer (300 mM, pH 3.6), a solution of 10 mM TPTZ in 40 mM HCl, and 20 mM FeCl_3_ × 6H_2_O at 10:1:1 (v/v/v). Firstly 300 μL of freshly prepared FRAP reagent was heated to 37 °C and an absorbance (A_0_) of a blank reagent was read at 593 nm in a Biotek EL808 microplate reader (Winooski, VT, USA). Then 10 μL of 0.01% 5,8-DHC solution in water and 30 μL H_2_O were added and the absorbance (A) was recorded every 1 min during the whole monitoring period which lasted up to 30 min. The change in the absorbance (ΔA_593_ nm) between the final reading and A_0_ was calculated for each sample and related to the ΔA_593_ nm of a Fe^2+^ reference solution which was measured simultaneously*.* The value is expressed in Trolox equivalents required to obtain similar reducing power. 

#### 3.2.5. Oil Oxidation Measurement in Oxipress Apparatus

The samples were prepared by mixing rapeseed oil with 0.025, 0.05 and 0.1% of DHC. Five g of oil were placed in a reactor tube and thermostated at 120 °C under oxygen atmosphere (5 bars) in Oxipress apparatus (Mikrolab, Aarhus). Pressure changes which occur due to the absorption of oxygen consumed for oil oxidation were recorded. The protection factor (PF) values of rapeseed oil in case of using 5,8-DHC were calculated by the following formula:

PF = IP_X_/IP_K_ (3)

where IP_X_ is induction period of sample with additive (h); IP_K_ is induction period of sample without additive (h). 

### 3.3. Chromosome Aberration and Micronucleus Assay in Wistar Rat Bone Marrow Cells *In Vivo*

In the present study, we evaluated two cytogenetic endpoints, such as CAs and MN, in the same animal. Rats are generally used for CA assessment, and rarely in the MN, mainly because granules from ruptured leukocytes are thought to resemble MN and contaminate bone marrow smears [52]. However, there are several reports on the concurrent analysis of CAs and MN in the same animal [53,54]. This approach has several advantages, first of all, such as reducing overall animal usage and correlating genotoxicity results from different endpoints.

#### 3.3.1. Animals and Treatment Schedule

Male albino Wistar rats aged 8–10 weeks and having body weight of 200–220 g, were used in the present study. The animals were supplied by the Animal Facility of the Department of Immunology, State Research Institute Center for Innovative Medicine. Ethical approval was given by the Committee of the Lithuanian Animal Care and Use. Prior to the start of the study, the animals were acclimatized for 7 days. They were housed under conditions of constant temperature, humidity and a light/dark cycle of 12 h/12 h with free access to standard commercial pellet diet and drinking water. The animals were randomly distributed into 11 experimental groups of six rats each.

We used a mixture of dimethylsulfoxide (DMSO) and sunflower oil (1:2) as the vehicle for 5,8-DHC (5,8-DHC was dissolved in DMSO and adjusted to the desired dose with sunflower oil immediately before administration). For the acute treatment, the animals received single intraperitoneal (i.p.) injections of 10 or 20 mg/kg of 5,8-DHC in 1 mL volume per 100 g body weight (b.w.). The negative control group comprised animals, given a single i.p. injection of vehicle (1 mL/100 g b.w.) and the positive control group, given cyclophosphamide (CP, 30 mg/kg adjusted in 0.5 mL/100 g b.w. in sterile physiological saline). A commercial form of cyclophosphamide was used (‘Endoxan’, ASTA Medica AG, Frankfurt, Germany). Blank controls comprised untreated animals. For the repeated treatment, 5,8-DHC (10 or 20 mg/kg) or vehicle was administered *via gavage* (1 mL/100 g b.w.) once a day for 3 consecutive days. Animals were sacrificed 24 h or 48 h after the last dose administration. Colchicine was applied i.p. at a dose of 2 mg/kg b.w. in 1 mL volume 90 min before sacrifice. Both femurs were dissected from each animal and used to obtain bone marrow preparations for the analysis of chromosome aberrations and micronucleus. 

#### 3.3.2. Chromosome Aberration *in Vivo* Assay

Bone marrow cells were flushed from the femora with 0.55% potassium chloride hypotonic solution, incubated for 25 min at 37 °C and then centrifuged at 150 × *g* for 8 min. Cells were fixed in methanol-glacial acetic acid (3:1). Slides were prepared by a flame-drying procedure and then stained with 5% Giemsa stain. Slides were coded and scored blind by the single scorer at a magnification of 1500× (Nikon ECLIPSE E200, Tokyo, Japan). Only well-spread metaphases with 42 ± 1 chromosomes were used for the analysis. The frequencies of CAs were estimated in 100 metaphases per animal. Aberrations were recorded as individual types according to J.R. Savage’s classification [55] but for convenience were grouped as chromatid breaks (ctb), chromatid exchanges (cte), chromosome breaks (csb) and chromosome exchanges (cse). Gaps were counted but not included into statistical analysis. 

#### 3.3.3. Micronucleus *in Vivo* Assay

The bone marrow MN assay was conducted according to the standard procedure [56]. Ends of the femura were cut off and the bone marrow was washed out with 2 mL of heat-inactivated foetal calf serum (Biochrom AG, Berlin, Germany), mixed thoroughly to obtain a fine suspension and centrifuged for 5 min at 150 × *g*. The supernatant was discarded and the cell pellet was carefully re-suspended. At least two smears per animal were prepared and allowed to air dry prior to fixation with methanol. The slides were stained in May-Grünwald solution followed by Giemsa (both Merck), coded and scored blind by the same scorer under magnification of 1000× (Jenaval, Zeiss, Jena, Germany). Micronucleated polychromatic erythrocyte (MNPCE) frequencies were based on the observation of 2,000 immature polychromatic erythrocytes (PCEs). Bone marrow toxicity was monitored by a decrease of PCE to total erythrocytes (e.g., PCEs+NCEs, normochromatic erythrocytes). At least 1,000 bone marrow erythrocytes per animal were analysed. 

### 3.4. Cytogenetic Test in Human Lymphocytes *in Vitro*

Blood samples were taken from two healthy female volunteers, 46 years old (donor A) and 45 years old (donor B). Whole peripheral blood was grown in HEPES-buffered RPMI 1640 medium supplemented with 12% heat-inactivated newborn calf serum, 7.8 μg/mL phytohemagglutinin P, 50 μg/mL gentamycin, 10 μg/mL 5-bromo-2'-deoxyuridine. All reagents used for the cell culture were purchased from Sigma-Aldrich Chemical Co. (St. Louis, MO, USA). Cell cultures were incubated at 37 °C for 72 h. Treatment with test compounds was carried out 48 h after culture initiation and lasted for the period of 24 h. Dose range of 5,8-DHC (10 to 40 µg/mL) was based on pilot experiments (data not shown). As whole blood cultures display many properties common with the liver microsomal cytochrome P450 system [57], no external metabolising enzymes were added. 5,8-DHC was dissolved in ethanol and then diluted with RPMI 1640 medium to the desired concentration. Ethanol in a final concentration of 7.5µL/mL was used as a solvent control. Ethanol concentration in the experimental series did not exceed this concentration. Working solutions were made just before treatment. Two parallel cultures were used for each concentration of the compound tested. Two cultures were left untreated and served as a blank control. Methyl methanesulfonate (MMS) in a final concentration of 0.02 μL/mL was used as a positive control. The cultures were exposed to colchicine at a final concentration of 0.6 μg/mL for the last 3 h of incubation. The cells were harvested, hypotonically swollen in 0.075 M KCl and fixed in methanol: acetic acid (3:1). Air-dried slides were differentially stained by fluorescence plus Giemsa technique as has been described previously [24]. Briefly, the slides were stained for 10 min. with 10 μg/mL of Hoechst 33258 dye (dissolved in 0.07 M Soerensen’s buffer, pH 6.8). Then the slides were rinsed, mounted with citrate buffer (pH 8.5), covered with cover slips and exposed to UV light (400 W mercury lamp at a distance of 15 cm) for 6–7 min. Slides were then rinsed and stained for 3–4 min. with 5% Giemsa. Cytogenetic analysis was performed on coded slides. No less than 100 first-mitotic division metaphases per culture were analysed for CAs, and no less than 50 second-division metaphases for SCEs.

Cell replicative kinetics were determined by means of a replicative index (RI = [M_1_ + 2M_2_ + 3M_3_]/N, where M_1_, M_2_, M_3_ are the numbers of cells that had undergone one, two or three cycles of replication, and N is a total number of cells scored). 300 cells were scored to determine RI 

### 3.5. Somatic Mutation and Recombination Test (SMART) in Drosophila Melanogaster *in Vivo*

The basic procedures for the *D. melanogaster* somatic mutation and recombination test (SMART) were performed according to Graf and co-workers [58,59]. Two *D. melanogaster* strains (kindly provided by Dr. H. Frei, Zurich, Switzerland) were used in the present study: virgin females from the strain *ORR/ORR; flr^3^/ In (3LR) TM3, ri p^p^ sep l(3)89Aa bx^34e^ e Bd^S^* were mated with *mwh*/*mwh* males. The ORR strain has chromosomes 1 and 2 from a DDT-resistant Oregon line, which are characterized by constitutively over-expressed *CYP450* genes. The CYP 450 enzymes play the main role in the bioactivation of xenobiotics, as well as natural products including phytochemicals [60]. Thus, the use of this high-bioactivation strain of *D. melanogaster* facilitates the detection of promutagens of numerous classes of compounds. The markers *mwh* (multiple wing hairs) and *flr^3^* (misshapen, flare-like hairs) are recessive wing-hair mutations located on the third chromosome at 0.3 and 38.8 respectively.

Eggs from the crosses were collected over 10 h periods. Progeny were raised on the Instant Drosophila Medium (Sigma Chemical Co., St. Louis, MO, USA) at 25 °C. Two schedules of the larvae treatment were applied. Firstly, the crossed flies were permitted to lay eggs for 10 h in vials containing medium prepared with 5,8-DHC solution. Thus, the exposure duration was about 120 h (*i.e.*, evolutionary period from fly egg till case-worm). According to the second schedule, the 72 ± 5 h-old larvae were exposed to 5,8-DHC by adding test solution to the surface of the medium and were fed on this medium for the rest of their development, during approximately 48 h. 5,8-DHC was dissolved in ethanol and then diluted with distilled water to the desired concentration, with the final ethanol concentration 5% v/v. The solutions were always prepared immediately before use. Solvent controls were included in all treatments. Methyl methanesulfonate was included in the assay as a positive control. Trans-heterozygous (*mwh flr^+^ / mwh^+^ flr^3^*) flies were collected and stored in a 70% ethanol. The wings of adult flies were mounted on glass slides in Faure’s solution, coded and scored at 400× magnification (Nikon E200) for the presence of single (*mwh* or *flr^3^* phenotype) or twin (adjacent *mwh* and *flr^3^* clones) spots. The spots were recorded according to standard procedures [58]. No less than 40 wings were analysed per each experimental point. Two repeated experiments were carried out.

### 3.6. Statistical Analysis

Statistical analyses were performed using InStat V2.02 (GraphPad Software, La Jolla, CA, USA) statistical package. Statistical tests were chosen according to the nature of the data analysed. *X^2^-*test with Yate’s correction was used to estimate results of CA in rat bone marrow cells *in vivo* and SMART assays. A one-way analysis of variance (ANOVA) and the Student’s two-sided *t-*test was used for the evaluation of SCE and CA in human lymphocytes *in vitro* and MN in rat bone marrow cells *in vivo*, and *z-*test [38] for RI analysis in human lymphocyte cultures. *p* < 0.05 was considered as the level of significance. 

## 4. Conclusions

In the present study, 5,8-DHC was shown to possess strong radical scavenging properties and antioxidant activity and thus could be considered as a potential antioxidant compound of natural origin. The genotoxicity data show that 5,8-DHC did not increase frequency of CAs in Wistar rat bone marrow cells, but induced a significant increase of MNPCEs. A possible aneugenic effect of 5,8-DHC *in vivo* merits further study. 5,8-DHC was slightly mutagenic in a *Drosophila melanogaster* assay *in vivo* after 120 h of treatment, but not after 48 h of treatment*.* 5,8-DHC induced both CAs and SCEs in human lymphocytes *in vitro* in a clear dose-dependent manner. Inter-donor variability in the number of 5,8-DHC-induced SCEs was observed. Thus, 5,8-DHC may be classified as weakly genotoxic both *in vivo* and *in vitro*. 

## References

[1] Lake B.G. (1999). Coumarin metabolism, toxicity and carcinogenicity: Relevance for human risk assessment. Food Chem. Toxicol..

[2] Hoult J.R.S., Paya M. (1996). Pharmacological and biochemical actions of simple coumarins: Natural products with therapeutical potential. Gen. Pharmacol..

[3] Bilgin H.M., Atmaca M., Obay B.D., Ozekinci S., Tasdemir E., Ketani A. (2011). Protective effects of coumarin and coumarin derivatives against carbon tetrachloride-induced acute hepatotoxicity in rats. Exp. Toxicol. Pathol..

[4] Kostova I. (2005). Synthetic and natural coumarins as cytotoxic agents. Curr. Med. Chem. Anticancer Agents.

[5] Voora D., McLeod H.L., Eby C., Gage B.F. (2005). The pharmacogenetics of coumarin therapy. Pharmacogenomics.

[6] Wu C.R., Huang M.Y., Lin Y.T., Ju H.Y., Ching H. (2007). Antioxidant properties of cortex Fraxinis and its simple coumarins. Food Chem..

[7] Pukalskas A., van Beek T.A., Venskutonis P.R., Linssen J.P.H., van Veldhuizen A., de Groot A. (2002). Identification of radical scavengers in sweet grass (*Hierochloe odorata*). J. Agric. Food Chem..

[8] Imran M., Riaz N., Ibrahim M., Ahmed E., Rasool M., Malik A., Moazzam M. (2009). Further phytochemical studies on *Aerva persica*. J. Chem. Soc. Pak..

[9] Bandonienė D., Pukalskas A., Venskutonis R., Gruzdienė D. (2000). Preliminary screening of antioxidant activity of some plant extracts in rapeseed oil. Food Res. Int..

[10] Zainuddin A., Pokorny J., Venskutonis R. (2002). Antioxidant activity of sweetgrass (*Hierochloe odorata* Wahlnb.) extract in lard and rapeseed oil emulsions. Nahrung.

[11] Augustyniak A., Bartosz G., Čipak A., Duburs G., Horáková L., Łuczaj W., Majekova M., Odysseos A.D., Rackova L., Skrzydlewska E. (2010). Natural and synthetic antioxidants: An updated overview. Free Rad. Res..

[12] Nemeikaitė-Čėnienė A., Marozienė A., Pukalskas A. (2005). Redox properties of novel antioxidant 5,8-dihydroxycoumarin: Implications for its prooxidant cytotoxicity. Z. Naturforsch. C. J. Biosci..

[13] EFSA (European Food Safety Authority) (2004). Opinion of the Scientific Panel on Food Additives, Flavourings, Processing Aids and Materials in Contacts with Food (AFC) on a request from the Commission related to coumarin. EFSA J..

[14] Api A.M. (2001). Lack of effect of coumarin on the formation of micronuclei in an *in vivo* mouse micronucleus assay. Food Chem. Toxicol..

[15] NTP (National Toxicology Program) (1993). Toxicology and carcinogenesis studies of coumarin (CAS No. 91–64–5) in F344/N rats and B6C3F1 mice (gavage studies). Natl. Toxicol. Program Tech. Rep. Ser..

[16] Lin H.C., Tsai S.H., Chen C.S., Chang Y.C., Lee C.M., Lai Z.Y., Lin C.M. (2008). Structure–activity relationship of coumarin derivatives on xanthine oxidase-inhibiting and free radical-scavenging activities. Biochem. Pharmacol..

[17] Lin W.L., Wang C.J., Tsai Y.Y., Liu C.L., Hwang J.M., Tseng T.H. (2000). Inhibitory effect of esculetin on oxidative damage induced by t-butyl hydroperoxide in rat liver. Arch. Toxicol..

[18] Huang D., Ou B., Prior R.L. (2005). The chemistry behind antioxidant capacity assays. J. Agric. Food Chem..

[19] Nakagawa H., Hasumi K., Woo J.-T., Nagai K., Wachi M. (2004). Generation of hydrogen peroxide primarily contributes to the induction of Fe (II)-dependent apoptosis in Jurkat cells by (D) - epigallocatechin gallate. Carcinogenesis.

[20] Galati G., O’Brien P.J. (2004). Potential toxicity of flavonoids and other dietary phenolics: significance for their prevention and anticancer properties. Free Rad. Biol. Med..

[21] Metodiewa D., Jaiswal A.K., Čėnas N., Dičkancaitė E., Segura-Aguilar J. (1999). Quercetin may act as a cytotoxic prooxidant after its metabolic activation to semiquinone and quinoidal product. Free Rad. Biol. Med..

[22] Boersma M.G., Vervoort J., Szymusiak H., Lemanska K., Tyrakowska B., Čėnas N., Segura-Aguilar J., Rietjens I.M.C.M. (2000). Regioselectivity and reversibility of the glutathione conjugation of quercetin quinone methide. Chem. Res. Toxicol..

[23] Mennen L.I., Walker R., Bennetau-Pelissero C., Scalbert A. (2005). Risks and safety of polyphenol consumption. Am. J. Clin. Nutr..

[24] Sakihama Y., Cohen M.F., Grace S.C., Yamasaki H. (2002). Plant phenolic antioxidant and prooxidant activities: Phenolics-induced oxidati*ve* damage mediated by metals in plants. Toxicology.

[25] Shukla Y., Arora A., Taneja P. (2002). Antimutagenic potential of cumarin on chromosomal aberrations in Wistar rats. Mutat. Res..

[26] Kocak N., Üstün H., Gülkaç M.D., Kanli A.Ö., Borazan A., Yilmaz A. (2004). Effect of 1α,25-dihydroxyvitamin D_3_ on doxorubicin-induced chromosomal aberrations in rat bone marrow cells. Acta Oncol..

[27] De Souza A.B., Souza L.M.S., Carvalo J.C.T., Maistro E.L. (2006). No clastogenic activity of *Caesalpinia ferrea* Mart. (Leguminosae) extract on bone marrow cells of Wistar rats. Genet. Mol. Biol..

[28] Espósito A.V., Pereira D.M.V., Rocha L.M., Carvalho J.C.T., Maistro E.L. (2005). Evaluation of the genotoxic potencial of the *Hypericum brasiliense* (Guttiferae) extract in mammalian cell system *in vivo*. Genet. Mol. Biol..

[29] De Mattos J.C.P., de Matos V.C., Rodriges M.P., de Oliveria M.B.N., Dantas F.J.S., Santos-Filho S.D., Bernardo-Filho M., Calderia-de-Araujo A. (2012). Evaluation of deoxyribonucleic acid toxicity induced by the radiopharmaceutical ^99m^Technetium-methylenediphosphonic acid and by stannous chloride in Wistar rats. Molecules.

[30] Hamada S., Yamasaki K., Nakanishi S., Omori T., Serikawa T., Hayashi M. (2001). Evaluation of the general suitability of the rat for the micronucleus assay: the effect of cyclophosphamide in 14 strains. Mutat. Res..

[31] Felter S.P., Vassallo J.D., Carlton B.D., Daston G.P. (2006). A safety assessment of coumarin taking into account species-specificity of toxicokinetics. Food Chem. Toxicol..

[32] Morris D.L., Ward J.B. (1992). Coumarin inhibits micronuclei formation induced by benzo(a)pyrene in male but not female ICR mice. Environ. Mol. Mutagen..

[33] Edwards A.J., Price R.J., Renwick A.B., Lake B.G. (2000). Lack of effect of coumarin on unscheduled DNA synthesis in the *in vivo* rat hepatocyte DNA Repair Assay. Food Chem. Toxicol..

[34] Swenberg J.A. (2003). Covalent Binding Index Study on Coumarin. Report of Laboratory of Molecular Carcinogenesis and Mutagenesis.

[35] Baskaran N., Rajasekaran D., Manoharan S. (2011). Coumarin protects 7,12-dimethylbenz(a)anthracene-induced genotoxicity in the bone marrow cells of golden Syrian hamsters. Int. J. Nutr. Pharmacol. Neurol. Dis..

[36] Madari H., Panda D., Wilson L., Jacobs R.S. (2003). Dicoumarol: A unique microtubule stabilizing natural product that is synergistic with taxol. Cancer Res..

[37] Podbielkowska M., Piwocka M., Waszkowska E., Waleza M., Zobel A.M. (1995). Effect of coumarin and its derivatives on mitosis and ultrastructure of meristematic cells. Int. J. Pharm..

[38] Lazutka J.R. (1991). Replication index in cultured human lymphocytes: methods for the statistical analysis and possible role in genetic toxicology. Environ. Mol. Mutagen..

[39] Galloway S.M., Armstrong M.J., Reuben C., Colman S., Brown B., Cannon C., Bloom A.D., Nakamura F., Ahmed M., Duk S. (1987). Chromosome aberrations and sister chromatid exchanges in Chinese hamster ovary cells: evaluation of 108 chemicals. Environ. Mol. Mutagen..

[40] Sasaki Y.F., Imanishi H., Ohta T., Shirasu Y. (1987). Effects of antimutagenic flavorings on SCEs induced by chemical mutagenesis in Chinese hamster cells. Mutat. Res..

[41] Sasaki Y.F., Imanishi H., Watanabe M., Ohta T., Shirasu Y. (1990). Suppressing effect of antimutagenic flavorings on chromosome aberrations induced by UV-light or X-rays in cultured Chinese hamster cells. Mutat. Res..

[42] Kaya B., Marcos R., Yanikoglu A., Creus A. (2004). Evaluation of the genotoxicity of four herbicides in the wing spot test of *Drosophila melanogaster* using two different strains. Mutat. Res..

[43] Rojas-Molina M., Campos-Sánchez J., Analla M., Munoz-Serrano A., Alonso-Moraga A. (2005). Genotoxicity of vegetable cooking oils in the Drosophila wing spot test. Environ. Mol. Mutagen..

[44] Tellez M.G.O., Rodriguez H.B., Olivares G.Q., Sortibran A.N.C., Cetto A.A., Rodriguez-Arnaiz R. (2007). A phytotherapeutic extract of *Equisetum myriochaetum* is not genotoxic either *in vivo* wing somatic test of *Drosophila melanogaster* or in the *in vitro* human micronucleus test. J. Ethnopharmacol..

[45] Würgler F.E., Vogel E.W., De Serres F.J. (1986). *In Vivo* mutagenicity testing using somatic cells of Drosophila *melanogaster*. Chemical Mutagens, Principles and Methods for their Detection.

[46] Graf U., Spano M.A, Rincon J.G., Abraham S.K., de Andrade H.H. (1996). The wing somatic mutation and recombination test (SMART) in *Drosophila melanogaster*: An efficient tool for the detection of genotoxic activity of pure compounds or complex mixtures as well as for studies on antigenotoxicity. Afr. Newslett. Occup. Health Safety.

[47] Pukalskas A. (2008). Isolation, Identification and activity of natural antioxidants from sweet grass (*Hierochloe odorata*), Costmary (*Chrysanthemum balsamita*) and horehound (*Marrubium vulgare*), cultivated in Lithuania. PhD Thesis.

[48] Brand-Williams W., Cuvelier M., Berset C. (1995). Use of a free radical method to evaluate antioxidant activity. LWT-Food Sci. Technol..

[49] Re R., Pellegrini N., Proteggente A., Pannala A., Yang M., Rice-Evans C. (1999). Antioxidant activity applying an improved ABTS radical cation decolorization assay. Free Radic. Biol. Med..

[50] Folin C., Ciocalteu V. (1927). Tyrosine and tryptophane determination in protein. J. Biol. Chem..

[51] Benzie I.F., Strain J.J. (1996). The ferric reducing ability of plasma (FRAP) as a measure of, antioxidant power”: the FRAP assay. Anal. Biochem..

[52] Mavournin K.H., Blakey D.H., Cimino M.C., Salamone M.F., Heddle J.A. (1990). The *in vivo* micronucleus assay in mammalian bone marrow and peripheral blood. A report of the US Environmental Protection Agency Gene-Tox Program. Mutat. Res..

[53] Krishna G., Theiss J.C. (1995). Concurrent analysis of cytogenetic damage *in vivo*: A multiple endpoin-multiple tissue approach. Environ. Mol. Mutagen..

[54] Slapsyte G., Mierauskiene J., Morkunas V., Prasmickiene G., Didziapetriene J. (2007). Modifying effects of sodium selenite on adriamycin and cyclophosphamide induced chromosome damage and changes of antioxidant status in rats. Trace Elem. Electroly..

[55] Savage J.R. (1976). Classification and relationships of induced chromosomal structural changes. J. Med. Genet..

[56] Schmid W. (1975). The micronucleus test. Mutat. Res..

[57] Starke D.W., Mieyal J.J. (1989). Haemoglobin catalysis of a monooxygenase-like aliphatic hydroxylation reaction. Biochem. Pharmacol..

[58] Graf U., Würgler F.E., Katz A.J., Frei H., Juon H., Hall C.B., Kale P.G. (1984). Somatic mutation and recombination test in *Drosophila melanogaster*. Environ. Mutagen..

[59] Graf U., van Schaik N. (1992). Improved high bioactivation cross for the wing somatic mutation and recombination test in *Drosophila melanogaster*. Mutat. Res..

[60] Van Iersel M.L., Verhagen H., van Bladeren P.J. (1999). The role of biotransformation in dietary (anti)carcinogenesis. Mutat. Res..

